# Prevalence of Anxiety, Depression, and Body Dysmorphic Disorders Among Dermatology Outpatients With Acne Vulgaris at a Public Hospital in Saudi Arabia

**DOI:** 10.7759/cureus.64917

**Published:** 2024-07-19

**Authors:** Khadijah H Muzaffar, Hadiza B Halilu, Baraatu A Dantata, Shawqi M Saati, Loai A Salah

**Affiliations:** 1 General Practice, Batterjee Medical College, Jeddah, SAU; 2 Dermatology, East Jeddah Hospital, Jeddah, SAU

**Keywords:** body dysmorphic disorder, dermatology life quality index, depression, anxiety, acne vulgaris

## Abstract

Background: Acne vulgaris is a chronic inflammatory skin condition that primarily affects the face, affecting a person's physical appearance. Anxiety, depression, and body dysmorphic disorder (BDD) are the three primary psychological conditions seen in dermatological patients. This study aimed to assess how prevalent anxiety, depression, and BDD in acne patients and the negative effect of acne on quality of life and self-esteem in dermatology patients.

Materials and methods: This cross-sectional study was done at the dermatology clinic in East Jeddah Hospital, Jeddah, Saudi Arabia. Data was collected using two pre-designed questionnaires. Part 1 contained demographic information and part 2 included four sets of questionnaires including the dermatology life quality index (DLQI), Rosenberg Self-Esteem Scale (RSES), Hospital Anxiety and Depression Scale (HADS), and Body Dysmorphic Disorder Questionnaire (BDDQ). Post-acne hyperpigmentation index (PAHPI), global scale for acne scar severity (SCAR-S), and LEEDS were used by the dermatologist to assess the patients’ acne severity, post-acne scars, and pigmentation conditions. Data were analyzed using the IBM SPSS Statistics for Windows, Version 26 (Released 2019; IBM Corp., Armonk, New York, United States).

Results: The majority of the participants were females (76%), Saudi Arabians (95%), students (58%), single (82%), and attended university (66%). The mean acne duration was 5.75 ± 4.58 years and 44% of the participants used social media for more than five hours per day. The participants scored high on the RSES (27.54 ± 3.05), indicating normal self-esteem. The HADS-A score was 11.14 ± 2.74, whereas the HADS-D score was 11.46 ± 1.78, indicating anxiety and depression symptoms. Their mean SCAR-S score is 4.38 ± 2.89. DLQI scores (6.04 ± 6.05) indicate that acne had a moderate effect on patients' quality of life. The percentage of positive BDD patients is significantly higher than those with negative BDD (p = 0.022). Furthermore, a significant association between PAHPI total score and SCAR-S (r = 0.48, p ≤ 0.001) and HADS-D (r = 0.39, p = 0.005) total scores.

Conclusion: The study focuses on how acne vulgaris affects patients' quality of life. The findings suggest that acne-related quality of life is positively associated with anxiety and depressive symptoms. This study provides clinicians with practical advice for implementing a more thorough management strategy for acne vulgaris.

## Introduction

Inflammation and irritation of the skin are signs of skin illnesses [1]. Skin conditions tend to have a higher incidence of associated mental health complications compared to other health issues [1]. On the other hand, having a psychological illness might exacerbate both the psychological and dermatological aspects of a medical condition, potentially leading to the development or recurrence of skin diseases [2,3]. This mutual aggravation is a significant concern, underscoring the intricate relationship between mental health and dermatological conditions [2,3]. Poor mental health has also been linked to a lack of compliance with medications and may potentially be a separate source of discomfort and impairment [3]. Due to their chronic nature, impact on self-image, and stigmatizing qualities, psychological morbidity may nevertheless be secondary to dermatologic illnesses [3]. Anxiety, depression, and body dysmorphic disorder (BDD) are the three primary psychological conditions seen in dermatological patients [2].

Acne vulgaris is a prevalent chronic inflammatory dermatological condition, impacting in excess of 80% of individuals worldwide [4]. It is estimated that approximately 90% of adolescents and 15% of adults experience this disorder [4]. Acne is typically found on the face wherein the notion of body image is heavily influenced by facial appearance [5]. Individuals with facial acne may experience considerable psychological disabilities [5]. Females are more likely to experience severe, chronic, or late-onset acne that causes scaring which increases the patient's stress and anxiety [6]. Irritation and "skin-picking" caused by stress can cause scarring, inflammation, and hyperpigmentation [6].

A previous study on depression in dermatology patients showed that a significant number of patients had depressive symptoms [7]. In another study, anxiety and depression were found to be the two most common mood disorders among dermatology patients, at 29% and 14%, respectively [2]. A separate study on BDD in dermatology patients showed that these patients' dissatisfaction with their looks persisted even after receiving dermatological treatment, which, in severe situations, may cause them to harm themselves or even take their own lives [8].

Despite substantial studies on the etiology, dangers, and treatment of acne vulgaris, little scientific attention has been paid to the psychological and emotional consequences of this condition. Furthermore, numerous research examined the prevalence and impact of anxiety, depression, and BDD as separate entities in relation to dermatological conditions. This study aimed to assess how prevalent anxiety, depression, and BDD in acne patients and also to study the negative effect of acne on quality of life and self-esteem in dermatology patients. This would result in a better understanding of the impact of this condition on patients' psychological state and well-being.

## Materials and methods

Study design

This is a cross-sectional study done at the dermatology clinic in East Jeddah Hospital, Jeddah, Saudi Arabia to determine the prevalence of anxiety, depression, and BDD among patients with acne vulgaris.

Ethical consideration

Ethical approval was obtained from the Research Ethics Committee of East Jeddah Hospital, Jeddah, Saudi Arabia (approval no. A01360; date of approval: September 9, 2022). Moreover, this study is in accordance with the Helsinki Declaration for human subjects. Electronic consent was obtained from the patients prior to their participation.

Study criteria

Included in this study were patients 18 years and above, diagnosed with acne vulgaris by the dermatologist. Patients outside the age range and those on psychiatric medications were excluded.

Procedure

Data was collected using two pre-designed questionnaires. One questionnaire was completed by the patients in Arabic and consisted of two parts. Part 1 gathered demographic information including age, gender, nationality, educational level, income, occupation, and marital status. Part 2 included four sets of questionnaires that evaluated the participants' psychosocial conditions. These questionnaires were completed by patients in the clinic after providing their electronic consent.

Assessment

The listed scales and indexes below are used in this study to assess anxiety, depression, and BDD among patients with acne vulgaris.

Dermatology Life Quality Index (DLQI)

The DLQI is a 10-question self-administered index used to assess how dermatological conditions affect life quality [9]. A DLQI score of 0-1 indicates no effect at all on the patient's life; a DLQI score of 2-5 indicates a small effect on the patient's life; a DLQI score of 6-10 indicates a moderate effect on the patient's life; a DLQI score of 11-20 indicates a very large effect on the patient's life; and a DLQI score of 21-30 indicates an extremely large effect on the patient's life [9].

Rosenberg Self-Esteem Scale (RSES)

A validated standardized survey is used as a one-dimensional tool capable of categorizing self-esteem levels as low or high. This scale has a possible range of 10 to 30, with scores below 15 indicating low self-esteem and scores ≥15 indicating normal self-esteem [10].

Hospital Anxiety and Depression Scale (HADS)

The HADS was used to assess a person's level of anxiety and depression. It is a valid self-assessment instrument used in outpatient clinics to evaluate the psychological status of non-psychiatric patients without addressing somatic symptoms. It consists of the anxiety subscale (HADS-A) and the depression subscale (HADS-D). The HADS instrument has 14 components (7 items for each subscale). Each item has four response options, which are scored with values ranging from 0 to 3, with 3 indicating the highest level of anxiety or depression. A total HADS-A or HADS-D score ≥8 of 21 points is consistent with the presence of symptoms of anxiety or depression. The reliability and validity of the Arabic version of the HADS, which was used in this study, were demonstrated by Terkawi et al. [11].

Body Dysmorphic Disorder Questionnaire (BDDQ)

A validated, brief, self-administered questionnaire derived from the DSM-V diagnostic criteria for BDD. The BDDQ is a five-item self-administered questionnaire, and all items of the tool had to be coded 3 to qualify for the diagnosis of BDD. The scale of answers ranged from 1 (absent) to 3 (threshold or true). The three items were added together to form one variable for BDD diagnosis. BDD was defined as a score of 4 [12,13].

The second questionnaire was filled by the dermatologist after a thorough examination of the patient's condition using the following scales.

Post-acne Hyperpigmentation Index (PAHPI)

A validated scoring system evaluating the post-inflammatory hyperpigmentation in patients with acne vulgaris. It consists of three characteristics: size (S), intensity (I), and number of lesions (N), where the total score is the sum of the three domains (S + I + N).

*Global Scale for Acne Scar Severity *​​​​​​​*(SCAR-S) *

To provide regional SCAR-S grades, this scale was independently applied to the face, chest, and back. At baseline, acne scars on the face, chest, and back were graded on a six-point scale: 0 for clear, 1 for almost clear, 2 for mild, 3 for moderate, 4 for severe, and 5 for extremely severe. For each patient, a composite scar score (overall SCAR-S) was calculated by adding the three regional scar scores (range: 0-15) [14].

Leeds

Leeds is a classification of acne vulgaris revised by Cunliffe 2003: The Leeds acne grading system assesses the severity of acne using Leeds scores ranging from grade 1 (least severe) to grade 4 (most severe) [15].

Sample size

The study included patients who agreed to participate in our study from August 17, 2022, to March 08, 2023. The sample size of 50 patients was calculated using the Raosoft online calculator.

Statistical analysis

Data were analyzed using the IBM SPSS Statistics for Windows, Version 26 (Released 2019; IBM Corp., Armonk, New York, United States). To determine the relationship between variables, qualitative data was expressed as numbers and percentages, and the chi-squared test (χ^2^) was used. Quantitative data was expressed as mean and standard deviation (mean ± SD), and non-parametric variables were tested using the Mann-Whitney and Kruskal Wallis tests. Correlation analysis was performed using Spearman's test, and a p-value of less than 0.05 was considered statistically significant.

## Results

This study included 50 participants of which 66% were aged 18 to 25 years old. The majority of the participants were females (76%), Saudi Arabians (95%), students (58%), single (82%), and attended university (66%). Results revealed that the mean acne duration was 5.75 ± 4.58 years and 44% of the participants used social media for more than five hours per day (Table 1).

**Table 1:** Distribution of the participants according to their demographic characters, acne duration, and daily use of social media (n = 50)

Variable	No. (%)
Age group	
18-25 years	33 (66)
26-35 years	14 (28)
36-45 years	3 (6)
Gender	
Female	38 (76)
Male	12 (24)
Nationality	
Non-Saudi	5 (10)
Saudi	45 (95)
Occupation	
Employee	13 (26)
Student	29 (58)
Unemployed	8 (16)
Marital status	
Single	41 (82)
Divorced	2 (4)
Married	7 (14)
Education	
Postgraduate	5 (10)
Secondary or less	12 (24)
University	33 (66)
Duration of acne (years)	5.75 ± 4.58
What is your daily use of social media?	
1-3 hours a day	8 (16)
3-5 hours a day	20 (40)
More than 5 hours per day	22 (44)

Table 2 shows the mean scores of the studied scales. Participants scored high on the RSES (27.54 ± 3.05), indicating normal self-esteem. However, it was also evident in HADS-A (11.14 ± 2.74) and HADS-D (11.46 ± 1.78) that participants exhibited symptoms of anxiety and depression. Some participants had severe acne scars on their faces, chests, and backs, as shown by their mean SCAR-S score of 4.38 ± 2.89. Additionally, DLQI scores (6.04 ± 6.05) indicate that dermatological conditions have a moderate effect on patients' lives.

**Table 2:** Mean and SD of studied scales (n = 50) DLQI: dermatology life quality index; PAHPI: post-acne hyperpigmentation index; SCAR-S: global scale for acne scar severity; HADS-anxiety score: Hospital Anxiety and Depression Scale-anxiety score; HADS-depression score: Hospital Anxiety and Depression Scale-depression score; RSES: Rosenberg Self-Esteem Scale; BDDQ: Body Dysmorphic Disorder Questionnaire

Scale	Mean ± SD
DLQI	6.04 ± 6.05
PAHPI	10.14 ± 3.93
SCAR-S	4.38 ± 2.89
HADS-anxiety score	11.14 ± 2.74
HADS-depression score	11.46 ± 1.78
RSES	27.54 ± 3.05
BDDQ	5.56 ± 3.15

No significant associations were found between DLQI and acne duration, use of social media, or patients’ demographics including age and gender. However, the acne duration of the patients with moderate or more effect (6.19 ± 5.66) on DLQI showed longer mean acne duration than those patients with a small effect on DLQI (5.43 ± 3.69). The SCAR-S classification revealed that 34.5% and 40% are practically clear for small and moderate effect groups, respectively. According to the HADS scale, 71% of the small effect group and 41.4% of the moderate effect group experienced anxiety, and 75.6% (small effect) and 66.7% (moderate effect) had depression. In the case of BDD, the percentage of positive BDD patients is significantly higher than those with negative BDD (p = 0.022).

Findings revealed that 15 out of 33 patients in the 18-25 age group have almost clear SCAR-S levels while only 2 of them have severe SCAR-S levels. There were no relationships identified between SCAR-S levels and patients' demographic characteristics, acne duration, or daily social media usage.

Spearman's correlation analysis found a significant association between PAHPI total score and SCAR-S (r = 0.48, p ≤ 0.001) and HADS-D (r = 0.39, p = 0.005) total scores. A non-significant correlation was established between the PAHPI total scores and the anxiety and depression domains of the HADS scale, the RSES, the BDDQ, or the DLQI scale scores (p ≥ 0.05) as shown in Table 3.

**Table 3:** Correlation between the PAHPI scale and SCAR-S, HADS-D total score, its anxiety and depression domains, and other scales PAHPI: post-acne hyperpigmentation; SCAR-S: global scale for acne scar severity; HADS-D: Hospital Anxiety and Depression Scale-anxiety score; HADS-anxiety score: Hospital Anxiety and Depression Scale-anxiety score; HADS-depression score: Hospital Anxiety and Depression Scale-depression score; RSES: Rosenberg Self-Esteem Scale; BDDQ: Body Dysmorphic Disorder Questionnaire; DLQI: dermatology life quality index

Variable	PAHPI total score	
	r	p-value
SCAR-S	0.48	<0.001
HADS-D total score	0.39	0.005
HADS-anxiety score	-0.008	0.957
HADS-depression score	0.18	0.192
RSES	-0.12	0.407
BDDQ	0.17	0.234
DLQI scores	0.14	0.321
Acne duration	0.09	0.513

## Discussion

Acne vulgaris is a chronic inflammatory skin condition that primarily affects the face, affecting a person's physical appearance [16]. Psychiatric co-morbidity is present in 40% of adult dermatological outpatients [17]. Acne, the most common skin condition of the second and third decades of life, has been linked with anxiety, rage, depression, and lower BDD [18]. Therefore, this could lead to tragic consequences if mental co-morbidity goes unacknowledged and undiagnosed.

In this study, we examined the physical characteristics of acne vulgaris in terms of severity, associated scarring, and pigmentation among dermatology patients at a public hospital in Saudi Arabia, and found its correlation with the psychological scales of HADS, BDD, RSES, and DLQI. The study also demonstrates the participant demographics, acne duration, and social media use in association with psychosocial scales. Unlike many studies that may focus on single aspects of psychological impact, this study integrates multiple psychometric scales. This holistic approach provides a more detailed understanding of the interplay between dermatological conditions and mental health.

The current findings revealed a higher prevalence of acne in females (76%) compared to males (16%) [19-21]. It's important to note that this study has a cross-sectional design, a small sample size, and is conducted at a single-center hospital [19-21]. Additionally, it is possible that female patients are more inclined to seek medical attention for acne-related concerns [19-21]. Therefore, while these results are consistent with recent studies in Bangladesh, Pakistan, and Saudi Arabia, indicating a higher prevalence of acne in females, they should be interpreted with caution [19-21]. Proper analysis and interpretation of the data are crucial to avoid overgeneralization or misinterpretation of the findings [19-21].

The study indicated that dermatological diseases have moderate effects on patients' quality of life, with a mean DLQI score of 6.04 ± 6.05. Previous investigations yielded similar outcomes. Hazarika and Rajaprabha's [22] hospital-based study in a suburban population in India found that the mean DLQI score was 7.22 and was statistically influenced by patients' age, duration, and grade of acne, acne scar, and post-acne. They also reported that DLQI increases with increasing age. In the cross-sectional study of Alkhezzi and Alotaibi [23], DLQI scores showed a mild effect on patient’s life in 34.2% of the study sample. There was no statistically significant correlation between the demographic factors and the 10 domains of the DLQI questionnaire.

It was discovered in this study that there is a significant relationship between DLQI and BDD. BDD is a psychological condition in which a person obsesses over physical imperfections. Positive BDD patients in this study have a moderate DLQI score which is somewhat similar to the studies conducted in Sweden and Saudi Arabia [8,24]. Dermatological disorders have an impact on patients' negative body image and quality of life, and this effect lasts even after dermatological treatment is completed. As demonstrated in a previous study, acne patients with BDD presented before treatment, diagnosed with even lower levels of overall satisfaction six months later [25]. AlShahwan [8] demonstrated that among Arab patients from the dermatology outpatient clinic, BDD was found in 14.1% with significant association with the female population and those who have hyperpigmentation or more than one skin condition [8].

HADS was utilized to evaluate the anxiety and depression of the patients in this study. In HADS-A, 5 of the patients were determined to be normal, 18 to be borderline abnormal, and 27 to be abnormal cases. The HADS depression assessment revealed that 1 was normal, 13 were borderline abnormal, and 36 were abnormal. According to the findings, patients with acne vulgaris showed symptoms of anxiety and depression, which may have a moderate impact on their quality of life. Previous studies reported that acne can negatively affect an individual’s psychological health, specifically causing rage, anxiety, and depression [19,26]. A study by Duman et al. (2015) showed that HADS-A and HAD-D were positively correlated with AQOL or the acne quality of life of patients [27].

A study done in a dermatology clinic in Riyadh emphasizes the importance of a multidisciplinary approach in the treatment of outpatients due to their high comorbidity with psychiatric disorders. Among Arab dermatology patients, anxiety and depression were prevalent (29% and 14%, respectively) [2].

The correlation of the SCAR-S scale for acne vulgaris showed that subjects with moderate to higher DLQI scores were found to be 47.6% with almost clear scars and 33.3% with mild scars. In an Egyptian study, it was reported that there is an association of acne vulgaris with depression and anxiety. Of the patients in the acne group who had severe acne, 44.4% had depression and 66.7% had anxiety [16]. In contrast, this study shows that even less severe forms can lead to substantial psychological outcomes, suggesting a need for early psychological intervention in acne treatment protocols, regardless of acne severity. In a study done in Makkah, the factors significantly associated with acne were age, gender, and severity. More than 50% of participants were female, whereas 12.3% of acne patients had severe depression and 28.5% had mild to moderate form of depression [17]. 

The study suggests that improving coping strategies for patients with moderate to severe acne severity can enhance their quality of life. The authors underline the importance of developing a positive attitude, which can reduce the likelihood of depression. Based on the findings, it might be more appropriate to include psychiatric consultation or psychotherapy in the treatment of moderate to severe acne vulgaris. Prompt intervention in acne vulgaris is crucial to preempt the onset of post-inflammatory hyperpigmentation and scarring, which can have profound psychosocial repercussions on patients. Dermatologists need to investigate the effect of acne vulgaris on quality of life and advise patients about available therapies.

The study's limitations included the cross-sectional design and convenient sample size therefore a causal relationship could not be demonstrated. In addition, this is a single-center hospital-based study with self-reported questionnaires. Although socioeconomic status, education level, and gender were assessed in the demographic assessments, future studies could benefit from a more in-depth exploration of these variables. To establish a more conclusive result, a larger sample size is recommended. Nonetheless, this study used validated Arabic-language questionnaires, and a dermatologist performed a thorough examination and diagnosis of acne vulgaris.

## Conclusions

The results of this study clearly demonstrate the impact of acne vulgaris on the quality of life of patients at the dermatology clinic in East Jeddah Hospital. The findings reveal that there is a significant correlation between quality of life and symptoms of anxiety and depression. Although the DLQI indicates that acne has a moderate impact on patients' lives, the high scores on the RSES suggest that despite experiencing symptoms of anxiety and depression, these patients maintain a generally healthy level of self-esteem. This suggests that while acne does affect aspects of daily life, it does not necessarily undermine the overall self-regard of the individuals suffering from it. To address these issues effectively, one possible solution is to regularly assess patients' anxiety and depression scores during follow-up visits. Based on these assessments, healthcare providers could refer patients for psychotherapy or initiate antidepressant therapy if needed. This proactive approach could help manage the psychological impact of acne, improving overall patient care and outcomes. This study provides practical guidelines for physicians to provide a more comprehensive management strategy for treating acne vulgaris.
